# Sodium Ions Affect GPR17 Conformational States and Functionality

**DOI:** 10.1002/prot.70157

**Published:** 2026-07-12

**Authors:** Luca Palazzolo, Simona Daniele, Davide Bianchi, Lara Russo, Stefano Capaldi, Maria Letizia Trincavelli, Ivano Eberini

**Affiliations:** ^1^ Dipartimento di Scienze Farmacologiche e Biomolecolari “Rodolfo Paoletti” Università degli Studi di Milano Milan Italy; ^2^ Department of Pharmacy University of Pisa Pisa Italy; ^3^ Department of Biotechnology University of Verona Verona Italy

**Keywords:** allosteric modulation, GPR17, radioligand binding, site‐directed mutagenesis, sodium ions

## Abstract

Sodium is an allosteric modulator of several class‐A GPCR regulating active versus inactive conformation state and may be a critical issue for the optimization of crystallization procedures. Herein, the role of Na^+^ in the regulation of GPR17 conformational states was studied through MD simulations of GPR17 wild‐type and mutated on two different residues relevant for sodium interaction (D77A and D293N), in inactive and active conformations. We observed that Na^+^ stabilizes the inactive wild‐type conformation more than the active conformation since in the latter the interaction between Na^+^ and D77/D293, fundamental in receptor stabilization, breaks. In addition to modeling studies, the role of sodium in GPR17 binding properties and functional responses was investigated in vitro using receptor variants carrying mutations at the sodium‐binding residues D77 and D293. Radioligand binding studies, using GPR17 selective ligands, did not show any significant alteration of GPR17 binding properties in mutated variants with respect to wild‐type receptor. In contrast, a significant increase in receptor coupling to G proteins was noticed for Asinex1 and LTD_4_ in the receptor variants D77, D293, and D77/D293 with respect to the wild‐type form. Finally, all the receptor ligands showed a significant increase in their potency to inhibit cAMP accumulation in D77, D293, and D77‐D293 GPR17 with respect to WT receptor form. Overall, these data, in agreement with modeling studies, suggest that ionic sodium stabilizes an inactive conformation of the receptor. When residues in the sodium‐binding site are mutated, the ion can no longer bind, thereby favoring an active conformation of GPR17. These mutagenized variants, stabilizing an active receptor state, may be the most suitable for homogeneous receptor purification and crystallization.

## 1 Introduction

GPR17, a class‐A GPCR, is expressed prominently in organs vulnerable to ischemic damage, such as the heart, kidney, and brain. This receptor holds significant promise as a therapeutic target for neurodegenerative diseases, notably multiple sclerosis (MS). Phylogenetically, GPR17 is related to the P2Y and cysteinyl‐leukotriene receptor families, positioning itself equidistantly between P2Y_12,13,14_ and the CysLTR receptor group [1].

GPR17 exhibits a dual pharmacological profile, interacting with extracellular uracil nucleotides (UDP, UDP‐glucose, and UDP‐galactose) and cysteinyl‐leukotrienes (LTC4, LTD4, and LTE4) during ischemic damage, where both classes of molecules are released at the site of inflammation [2].

Pharmacologically, GPR17 activation mediated by uracil nucleotides can be inhibited by P2Y‐R antagonists like cangrelor and MRS2179, while activation by cysteinyl‐leukotrienes can be suppressed by montelukast and pranlukast, which are CysLTR antagonists [2, 3, 4, 5, 6, 7, 8, 9]. GPR17 also responds to emergency signals like oxysterols, suggesting its promiscuous response to different signaling molecules based on physiological and pathological conditions. Furthermore, GPR17's response can change based on its dimerization with other receptors, including CXCR2 and CXCR4 [9].

Research efforts have led to the identification of novel GPR17 ligands from distinct chemical classes unrelated to previously identified derivatives. Cross‐talk mechanisms with chemokine receptors (CXCRs) have been explored, revealing that GPR17 can be activated by SDF‐1 (Stromal cell‐derived factor 1 or CXCL12), the cognate ligand for CXCR4 and CXCR7, with potency comparable to its activation of CXCRs.

Recent structural work captures GPR17 in an active, ligand‐free state bound to Gi, revealing a tethered‐agonist mechanism in which the 21‐residue extracellular loop 2 (ECL2) folds as a hairpin and penetrates the orthosteric pocket [10]. This segment is pinned by the C209–C132 disulfide and hydrogen bonds from ECL2 (Y213/R214) to TM7 (N307/R308), and it couples into the canonical activation network, driving TM6 displacement and Gi engagement. Within the helical core, a conserved sodium cavity centered on D77 lies immediately beneath the pocket and communicates with TM3/TM7 via a water‐mediated polar network. By analogy with class‐A GPCRs, Na^+^ occupancy of this site is expected to stabilize inactive, hydrated conformations, whereas activation should dehydrate/collapse the cavity. On this structural basis, this study delves into the role of sodium ions as regulators of GPR17 activity, aiming to deepen the understanding of the molecular mechanisms underlying the receptor functionality. Previous literature suggests that sodium ions can modulate several class‐A GPCRs by binding to a specific site in the helical core of the receptor. Mutagenesis experiments have identified the residue D77 (or D^2.50^ based on Ballesteros‐Weinstein numbering) as critical in mediating the effects of sodium on receptor activity, suggesting that the ion modulates receptor behavior by binding to a specific site [10].

High‐resolution crystallographic studies have confirmed the presence of a conserved sodium‐binding site in several class‐A GPCRs, with residue D^2.50^ playing a pivotal role in its coordination. Functionally, sodium ions are implicated in modulating the conformational equilibrium between active and inactive receptor states. The proposed mechanism involves sodium binding at a well‐defined site, where it forms a cluster with water molecules that stabilizes the inactive conformation and negatively modulates agonist binding. Receptor activation induces the collapse of this cluster and the formation of new hydrogen bonds that promote stabilization of the active state [10].

To explore the role of sodium ions in GPR17, we investigated three receptor conformations: the self‐activated *apo* state, the inactive *apo* state, and the active *holo* state in complex with the agonist galinex [6]. For each conformation, we examined the wild‐type receptor and three mutant variants: two single‐point mutants (D77A or D293N) and the corresponding double mutant (D77A + D293N).

## 2 Material and Methods

### 2.1 Computational Workflow

Our computational workflow included: (i) the combination of the available GPR17 cryo‐EM structure with homology modeling and molecular docking to generate the three conformational states; (ii) in silico alanine scanning to construct the mutant models; (iii) molecular dynamics (MD) simulations to equilibrate all systems; (iv) three‐dimensional reference interaction site model (RISM‐3D) analysis to identify and characterize ion‐binding sites (Figure 1).

**FIGURE 1:**
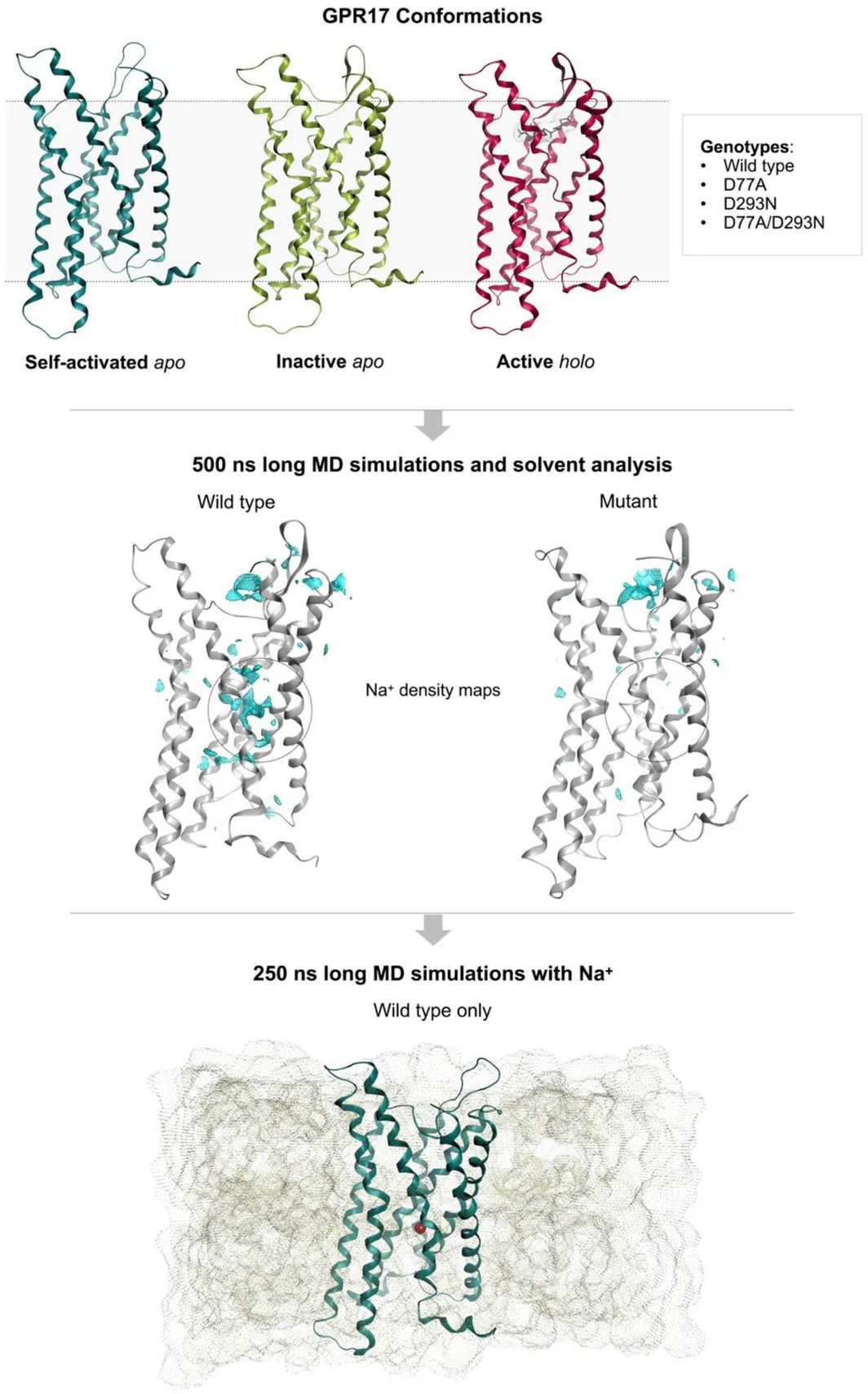
Experimental workflow of the in silico pipeline.

This integrated strategy enabled a detailed investigation of the molecular determinants underlying sodium binding to GPR17, providing novel insights into the structural basis of receptor modulation by monovalent cations.

Moreover, to confirm the effects of sodium ions on GPR17 binding properties and functionality, radioligand binding and functional assays were performed on 1321 N1 cells transfected with wild type GPR17 and the three mutated genotypes (single mutants, D77A or D293N; double mutant, D77A + D293N).

### 2.2 Homology Modeling

The experimentally solved structure of human GPR17 (PDB ID: 7Y89) [10] was used as a *self‐activated apo conformation*. It revealed key structural characteristics: the extracellular loop 2 (ECL2) occupies the orthosteric binding pocket of the receptor, and transmembrane helix 6 (TM6) adopted an active conformation. During activation of class‐A GPCRs, TM6 undergoes a substantial outward displacement on the cytoplasmic side facilitated by the disruption of the conserved *ionic lock*. This movement creates a binding cavity for the Gα subunit and is a hallmark conformational change underlying G protein engagement and signal transduction. Moreover, ECL2 plays a key role in ligand specificity and receptor activation by partially covering the orthosteric site and directly contributing to ligand recognition through specific residue interactions. Stabilized by a conserved disulfide bond with TM3, ECL2 can also participate in allosteric communication between ligand binding and intracellular conformational rearrangements. Accordingly, to investigate the role of sodium ions on specific GPR17 conformations, homology modeling techniques were employed to model the ECL2 in an “open” conformation and the TM6 helix in an inactive state.

The solved GPR17 structure was subjected to several preliminary steps using the Molecular Operating Environment (MOE) software (Version 2022, ChemComp Group, Quebec, Canada) to prepare the structure. These included the removal of the heterotrimeric G_i_ protein complexed with the receptor, the addition of caps on the N‐ and C‐termini, and the adjustment of the overall charge of the protein to ensure system neutrality.

Subsequently, a multiple sequence alignment was performed comparing phylogenetically related sequences from various class‐A GPCRs, including P2Y, cysteinyl‐leukotriene (CysLT), and chemokine (CXC) receptor families, including GPR17. Sequences were selected based on the presence of two conserved cysteines located in the N‐terminus and ECL3, which participate in the formation of a disulfide bond, missing in other class‐A GPCR subgroups. This alignment process was performed using the T‐coffee [11] (*Tree‐based Consistency Objective Function For Alignment Evaluation*) server, specifically the PSI/TM‐Coffee [11] version optimized for transmembrane proteins.

Based on the aligned sequences, the crystal structure of P2Y_1_ receptor complexed with the antagonist MRS2500 (PDB ID: 4XNW) [12] was chosen as a template.

In 4XNW, P2Y_1_ exhibited an inactive conformation of TM6 helix and an “open” conformation of ECL2. The template was refined by removing the associated rubredoxin and by solving structural problems using the “QuickPrep” tool provided by the MOE software. Finally, the first intracellular loop (ICL1) was modeled using the Loop Modeler tool, as it had been removed in the original structure due to crystallographic engineering involving rubredoxin fusion.

Then, after superposing the previously refined structure of GPR17 with 4XNW, the 3D structure of GPR17 in the inactive *apo* conformation was generated using the MOE “Homology Model” tool, modeling ECL2 in an “open” conformation and TM6 helix in an inactive state and overriding the model with the rest of GPR17 from 7Y89. The same approach was also used to create an active *apo* conformation for molecular docking simulations.

The obtained models were carefully checked with the MOE Protein Geometry module to verify that the stereochemical quality of the proposed structure was good.

### 2.3 Molecular Docking Simulations

The binding site of GPR17 in active *apo* conformation was identified through the MOE's “Site Finder” application that calculates possible active sites in a receptor from its 3D atomic coordinates using a geometric approach. This application generates a set of “dummy atoms” that mimic the potential location of a ligand within the binding pocket of the receptor.

This simulation, carried out using the “Dock” program of the MOE “Compute” module, was aimed at studying the interaction between GPR17 and the agonist named galinex.

The docking “Rigid Receptor” protocol was applied with a Triangle Matcher placing methodology. During this phase, 30 poses were obtained by aligning ligand triplets of atoms on triplets of receptor site points represented as alpha spheres, and they were ranked using the London dG scoring function that estimates the approx. binding free energy for the ligand from a given pose.

The refinement phase enabled the ligand to explore a vast array of conformations relative to the receptor, whose side chains remain rigid. In this phase, the GBVI/WSA dG empirical scoring function was applied to evaluate the binding free energy of the ligand from each pose. This process resulted in the selection of five final poses for further analyses. During these procedures, the AMBER10:EHT force field was used. The top scoring galinex pose was selected to build the active *holo* conformation of GPR17. Figure 2 resumes how the three GPR17 conformations were obtained.

**FIGURE 2:**
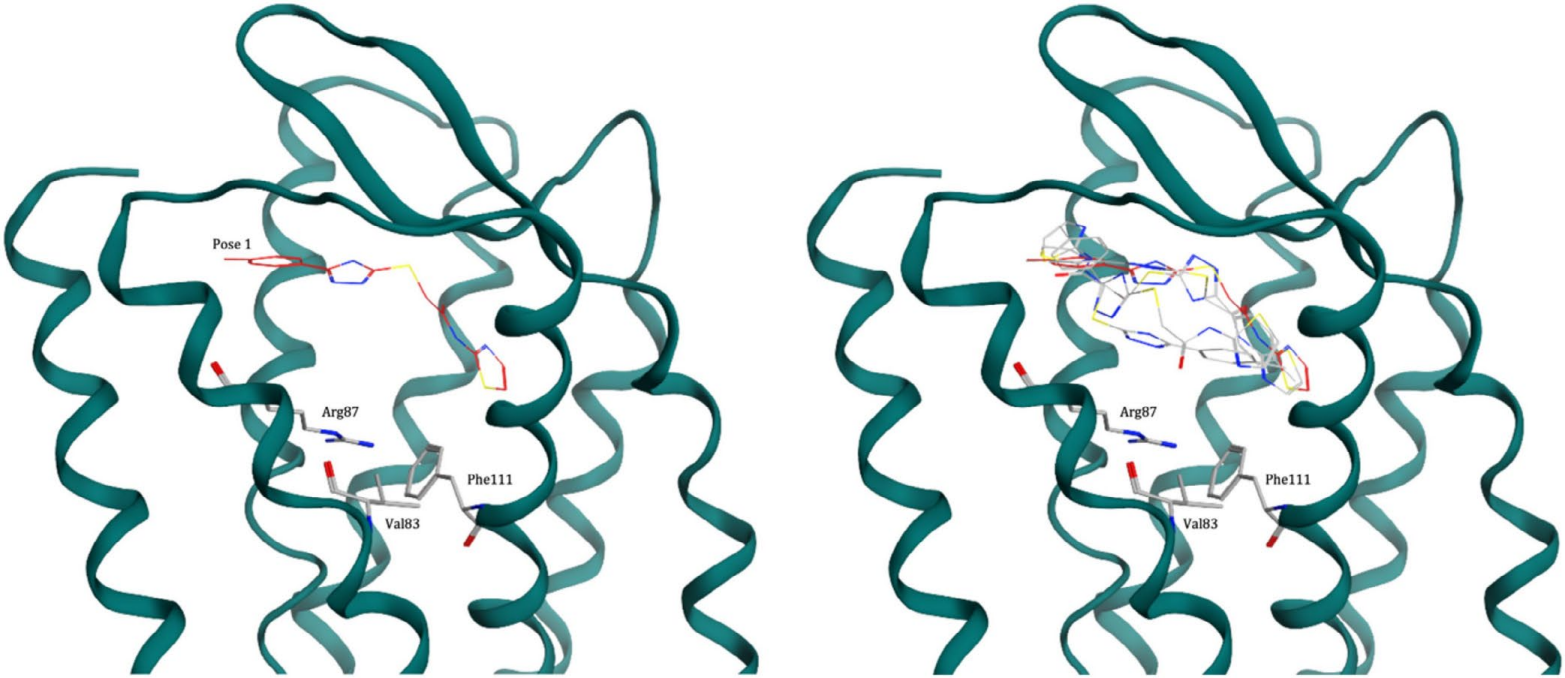
The top scoring (left) and the five best poses (right) resulting from the molecular docking simulations interacting with the orthosteric binding pocket of the receptor. The residues of the binding pocket (Val83, Arg87, and Phe111) are highlighted.

### 2.4 Residue Scan

Residue scan was performed using the corresponding module in MOE, with low‐mode molecular dynamics simulations employed to generate conformational ensembles for each mutant. Residue D77 was mutated into alanine in all receptor conformations to neutralize the negative charge on transmembrane TM2 helix. Position 7.49 in the Ballesteros–Weinstein numbering scheme is highly conserved among class‐A GPCRs, where it is most frequently occupied by asparagine (approx. 86%), while aspartic acid (D^7.49^) is present in only approx. 7% of receptors. The presence of D^7.49^ in GPR17 therefore represents a relatively rare, yet potentially functionally significant, variant. Given its putative role in sodium ion coordination and receptor activation, D293 (corresponding to D^7.49^) was mutated into asparagine in all conformations.

In addition to these two single‐point mutations, a double mutant (D77A + D293N) was generated for each receptor conformation, yielding a total of 12 distinct GPR17 structural models (Table 1).

**TABLE 1:** GPR17 conformations and genotypes used for the MD simulations.

GPR17^wt^	GPR17(D77A)	GPR17(D293N)	GPR17(D77A/D293N)
*apo* self activated	*apo* self activated	*apo* self activated	*apo* self activated
GPR17^wt^	GPR17(D77A)	GPR17(D293N)	GPR17(D77A/D293N)
*apo* inactive	*apo* inactive	*apo* inactive	*apo* inactive
GPR17^wt^	GPR17(D77A)	GPR17(D293N)	GPR17(D77A/D293N)
*holo* active bound to galinex	*holo* active bound to galinex	*holo* active bound to galinex	*holo* active bound to galinex

### 2.5 MD Simulations

Twelve MD simulations were performed, one for each of the 12 GPR17 structures (Table 1), using the software Amber22 [13]. The aim of these simulations was to assess the stability of the systems and to extract equilibrated, representative conformations, which are essential for carrying out further in‐depth analyses.

The initial phase of the study was focused on running 500 ns MD simulations of the different conformations of GPR17 across different genotypes. Through the “packmol‐memgen” module of AmberTools [13], the proteins were placed within a lipid bilayer composed of POPC according to their orientation in membrane computed by the PPM 2.0 server of OPM database (Orientation of Proteins in Membranes) [14]. The system was solvated using the OPC water model, adding Na^+^ and Cl^−^ at a concentration of 0.15 M. Periodic boundary conditions were set.

Following system setup, energy minimization was performed using the Newton–Raphson method, followed by the conjugated gradient method for further refinement. Once the minimization step occurred, the temperature of the system was increased from 0 to 300 K. The equilibration phase was performed in ensemble NPT, where pressure is kept constant using the Berendsen barostat first, and the Monte Carlo barostat later. The production phase was performed in NPT ensemble using the Monte Carlo barostat to control pressure and the Langevin thermostat to maintain a constant temperature. The simulations were performed using the following force fields: ff19SB for proteins, Lipid21 for lipids, TIP3P for water molecules and for Na^+^ and Cl^−^ ions, and gaff2 for other organic molecules, particularly for simulations involving GPR17::galinex complexes.

To evaluate system stability and conformational dynamics, several structural analyses were performed on the MD trajectories. The root mean square deviation (RMSD) of α‐carbons was used to monitor overall structural stability, while the root mean square fluctuation (RMSF) of backbone atoms assessed local flexibility. Hydrogen bond formation and persistence were geometrically analyzed to track interaction stability. Solvent‐accessible surface area (SASA) was calculated to estimate protein exposure to solvent, providing insight into folding and stability. Finally, cluster analysis based on RMSD profiles was conducted to identify representative conformational states, using hierarchical clustering of structurally similar frames.

All the analyses were performed using the CPPTRAJ tool of AmberTools and MDTraj, employing a hierarchical clustering algorithm (agglomerative clustering) for cluster analyses.

### 2.6 Solvent Analysis

The MOE Solvent Analysis application uses the 3D‐RISM method to analyze the role of solvent in macromolecular systems. The application computes a time‐averaged distribution of water H and O densities, along with free‐energy maps for analyzing solvent stability and solvation contribution to binding free‐energy. A unique feature of 3D‐RISM is its ability to include various concentrations of salt or hydrophobic molecules as part of the solvent, thereby producing density maps for ions and hydrophobic groups as well as for water.

Through this method, the structure of water can be described by calculating the time‐averaged spatial distribution of the solvent atoms. The particle densities reflect the spatial distribution of solvent atoms, which in turn characterizes the organization of solvent around a solute in a thermodynamic fashion. The analyses were performed using the AMBER10:EHT force field.

Starting from the results of the cluster analysis, the medoids of the most representative clusters of each simulation were submitted to solvent analysis, performed in “Solute” mode to calculate 3D solvent occupancies and free energy of solvation maps of a single collection of molecules (the solute) with no distinction made between ligands and receptors.

To limit the analysis to a specific region of the receptor, the amino acids of the transmembrane helices oriented toward the helical core were manually selected and set as “Near,” whereas all the receptor residues were selected and set as “Atoms.”

Each medoid was then analyzed in three different saline concentrations: 100 mM, 500 mM, and 1 M. According to the solvent analysis results, one Na^+^ atom was manually placed close to the GPR17 binding site, at the highest predicted density. Three GPR17∷ions complexes were built, one for each of the three conformations of GPR17^wt^. These complexes were then submitted to a 250 ns MD simulation following the previously described protocol.

### 2.7 Constructs Design and Preparation

The coding sequence of human GPR17 (short isoform, Uniprot ID Q13304‐2), without the stop codon, was amplified by PCR with primers designed to introduce the BamH I and Xho I restriction sites at the 5′ and 3′ termini of the ORF, respectively. The amplicon was digested with the restriction endonucleases and ligated into a pcDNA3.1/myc‐His A vector. The resultant construct allows the transient expression in mammalian cells of the wild‐type receptor with a C‐terminal myc epitope followed by a 6XHis tag. The conserved aspartic acid residues involved in the allosteric sodium binding (D^2.50^ and D^7.49^ according to the Ballesteros–Weinstein numbering, corresponding to D77 and D293 in GPR17) were identified in the GPCRbd database (https://gpcrdb.org/) and mutated to Ala and Asn respectively using the QuikChange site‐directed mutagenesis kit (Stratagene). In addition, a double mutant lacking both the acidic residues, D77A/D293N, was similarly prepared.

The GPR17‐BRIL 16–339 construct was cloned into the pFastBac CT‐Mouse vector, which was used to produce the recombinant bacmid according to the standard protocol for the Bac‐to‐Bac TOPO expression system (Life Technologies). All constructs were verified by DNA sequencing.

Expression and purification of the engineered GPR17 construct from insect cells was performed as described in Capelli et al. [15] with minor modification, Briefly, suspension cultures of ExpiSF9 cells (Life technologies) were grown at a density of ~3 × 10^6^ cells/mL and infected with high titer baculovirus stocks at moi of 3. Cells were collected 48 h post infection, resuspended in ice‐cold Lysis Buffer (10 mM HEPES, pH 7.5, 10 mM MgCl_2_, 20 mM KCl) supplemented with DNAse (20 μg/mL) and protease inhibitors and disrupted with a Dounce homogenizer at 4°C. The membrane fraction was collected by ultracentrifugation (45 000 rpm) for 45 min and solubilized with 0.5% β‐DDM. After centrifugation, the soluble fraction was loaded onto a SterpTactin TX column (IBA Lifesciences). After extensive washing with 0.1 M Tris‐Cl, 150 mM NaCl, 1 mM EDTA, pH 8 and 0.03% β‐DDM, the protein was eluted with the same buffer supplemented with 50 mM biotin. The protein was further purified by Immobilized Metal Affinity Chromatography (IMAC) on a Nikel‐NTA column and concentrated with 100 Kda cutoff concentrators (Millipore).

### 2.8 Cell Culture and Transfection

Astrocitoma cells (1321 N1) were grown in monolayer and maintained in Dulbecco's modified Eagle's medium F12 (DEMEM F12), supplemented with 10% (v/v) FBS, 100 U/mL penicillin, 100 μg/mL streptomycin at 37°C with 5% CO_2_ atmosphere until confluent.

For transfection, cells were seeded at a confluence of 24 500 cells/cm^2^ in a 24 wells plate (for binding assay and for cAMP assay) or in a 150 cm^2^ petri dish (for the [^35^S]GTPγS binding assay), overnight. The following day, cells were transfected with the jetPEI (Polyplus) transfection reagent according to the manufacturer's protocol. The mix was used on the cells and incubated 48 h in complete medium.

### 2.9 [ ^3^OH] 27‐Hydroxycholesterol Binding Assay

After transfection with GPR17 plasmide, 1321 N1 cells, cultured in 24 well multiplate, were fixed with 4% PFA and washed with HBSS‐Tween Buffer (8 g/L NaCl, 0.4 g/L KCl, 0.03 g/L Na_2_HPO4, 0.35 g/L NaHCO_3_, 0.06 g/L KH_2_PO_4_, 0.005% Tween) twice. Preliminary association kinetic experiments were performed by incubating the cells with the radioligand (0.5 nM) for different times (0–180 min). Moreover, saturation experiments in WT GPR17 cells were performed using different radioligand concentrations (ranging from 0.05 nM to 18 nM) to calculate [^3^H]27‐Hydroxycholesterol Kd value.

To determine the binding affinity of the GPR17 ligands, the cells were then treated with different concentration of LTD_4_ (0.5–100 nM), UDP‐glucose (1–100 μM) or Asinex1 (0.01 nM–10 nM) in presence of 0.5 nM of the radiolabeled [^3^H]27‐Hydroxycholesterol, and incubated for 2 h under shaking at room temperature. After incubation time, cells were washed and lysed with 200 μL of 0.1 M NaOH for 2 h followed by the addition of 200 μL of 0.1 M HCl to neutralize the solution. The lysate was collected in vials with a scintillation cocktail (Ultima Gold MV, PerkinElmer) and read at a scintillator counter (TRI‐CARB 2900TR, PerkinElmer). DPM values were analyzed using GraphPad Prism. Cells transfected with the empty vector were run in parallel as negative controls. The 100% reference corresponds to the DPM signal of [^3^H]27‐hydroxycholesterol measured in GPR17‐transfected cells that were not exposed to any unlabeled (cold) ligand.

### 2.10 [ ^35^S] GTPγS Binding Assay

In order to obtain cell membranes, transfected cells were lysed in a glass potter homogenizer with the lysis buffer (5 mM Tris·HCl, 2 mM EDTA, pH 7.4). The lysate was centrifuged at 48000 × g for 20 min at 4°C, and each pellet was rinsed with wash buffer (50 mM Tris·HCl, 10 mM MgCl_2_, pH 7.4) and homogenized. Proteins were quantified (Bradford assay), and membrane aliquots were stored at −80°C until used.

For the measurement of the functional response of the GPR17 receptor variants, [^35^S] GTPγS binding assay was performed as previously described [16] Briefly, in a filtering microplate (Merck Millipore MultiScreen GFB Filter Plates), 10 μg of membranes from WT, D77A, D293N, or D77A/D293N GPR17 were dissolved in binding buffer (20 mM HEPES, 3 mM MgCl_2,_ pH 7.4) and incubated with increasing concentrations of GPR17 agonists (UDP‐glucose, LTD_4_, or Asinex), or GPR17 antagonist, cangrelor, in the presence of 150 mM NaCl. After 15 min, [^35^S] GTPγS (0.2 nM) was added, and samples were incubated for 30 min at 30°C. Reactions were stopped by rapid filtration under vacuum. The filters were washed four times with binding buffer, removed from the multiplate, poured in a scintillation cocktail, and read at a scintillator counter (TRI‐CARB 2900TR, PerkinElmer). Cells transfected with the empty vector were run in parallel as negative controls. Data were analyzed using GraphPad Prism. All binding data were normalized to the basal radioactivity signal measured in membranes from GPR17 transfected cells not exposed to any ligand; this unstimulated DPM value was set to 100% in each independent experiment. Empty vector membranes were run in parallel as negative controls and did not show any significant specific binding or agonist induced stimulation. EC_50_ values were obtained from non‐linear regression of each single experiment, combined and expressed as mean value ± SEM. Data were tested for statistical significance using one‐way ANOVA versus the control (wild type GPR17). A value of *p* < 0.05 was considered significant.

### 2.11 cAMP Assay

Intracellular cAMP levels were tested by a radioactive binding assay.

On the assay day, 1321 N1 cell medium was replaced with a stimulation buffer containing a phosphodiesterase inhibitor (RO 20–1724) for 15′. After that, cells were treated with several Asinex1 (GPR17 agonist) concentrations for 15′ in the presence of 10 μM forskolin (cAMP activator).

For the radioactive assay, cells were washed in PSB, lysed with HCl for 30 min at room temperature and then neutralized with KOH. cAMP binding was measured incubating lysates with 2 nM [^3^H] cAMP (23.4 Ci/mmol). Nonspecific binding was measured, vs. the total, in the presence of an excess (0.166 mM) unlabeled ice‐cold cAMP. The reaction was terminated by rapid filtration through glass microfiber filters (Grade GF/D, Whatman, Merck), previously soaked in Tris HCl 50 mM, using a M48 cell harvester (Brandel Instruments, Gaithersburg, MD, USA). Filter were poured in a scintillation cocktail and read at a scintillator counter (TRI‐CARB 2900TR, PerkinElmer). The 100% reference corresponds to the maximal pmol cAMP per well produced by the non‐selective adenylate cyclase activator, forskolin, in each experiment; cAMP values in all wells were normalized to this forskolin‐induced maximal production. Data were analyzed using GraphPad Prism. Data were tested for statistical significance using one‐way ANOVA versus the control (wild type GPR17). A value of *p* < 0.05 was considered significant.

### 2.12 Statistical Analysis

Statistical analyses were performed using GraphPad Prism version 6.0 for Windows (GraphPad Software, San Diego, CA, USA). Data were subjected to One‐Way analysis of variance for mean comparison, and significant differences among different treatments were calculated according to Dunnett's multiple comparison test. Data are reported as mean ± SEM.

Differences at *p* < 0.05 were considered statistically significant.

## 3 Results

### 3.1 Homology Modeling

We used a homology modeling approach to generate 3D structures of GPR17 in both active and inactive conformations with ECL2 in the “open” configuration.

To validate the geometry of each structure, we checked the Ramachandran plot, confirming the overall quality of both models, as the only outlier values detected were associated with residues located in highly flexible regions of the proteins. Specifically, S90 is in the ICL1 while Y213 is in the ECL2 (Figure S1).

### 3.2 Molecular Docking

The molecular docking simulation showed that the agonist galinex interacts with the orthosteric binding pocket of GPR17 (Figure 2). This binding region includes the key residues V83, R87, and F111 [17], and it is notably distant from the sodium binding site, which includes D77 and D293, deeply located in the helical bundle.

The scores obtained for the five poses resulting from the refinement phase, as calculated by the GBVI/WSA dG scoring function, span between −6.78 and −6.56 kcal/mol with a complete overlap of functional groups among poses.

Based on these results, we chose to focus our subsequent analyses on the GPR17::galinex complex associated with the lowest docking score (−6.78 kcal/mol), as it represents the most probable pose and potentially holds the most biologically relevant binding modes.

### 3.3 MD Simulations

Twelve molecular dynamics simulations were performed on the different conformations of GPR17 across different genotypes with the aim to assess system stability and to obtain representative, equilibrated structures for subsequent analyses.

During the production phase of the simulation, the stability of the different systems was studied by calculating descriptors such as the RMSD, computed on the α‐carbons of the protein. This calculation revealed that all the systems reached equilibrium after approximately 200 ns of simulation, indicating that they achieved a conformational equilibrium following an initial adjustment phase. In all simulations, RMSD values remained below 4 Å throughout the simulated time (Figure 3).

**FIGURE 3:**
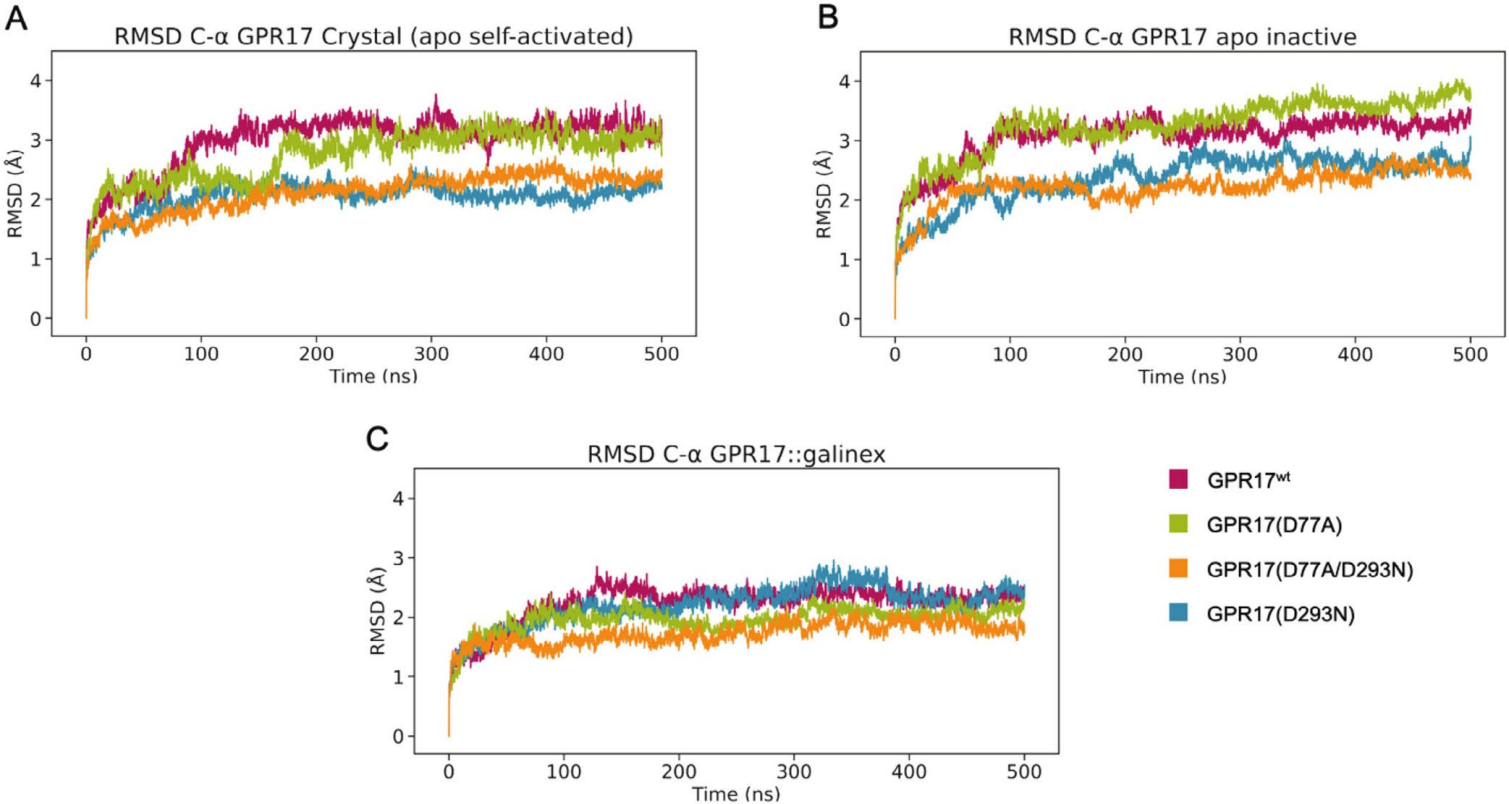
RMSD calculated on the α‐carbons of the protein in the different conformations and genotypes (red line: Wild type; green line: D77A; blue line: D293N; orange line: D77A/D293N): (A) *apo* active state, (B) *apo* inactive state, (C) *holo* active state.

Additional RMSD calculations focusing on galinex atoms were performed, where possible, to evaluate its structural stability over the simulation time (Figure 4).

**FIGURE 4:**
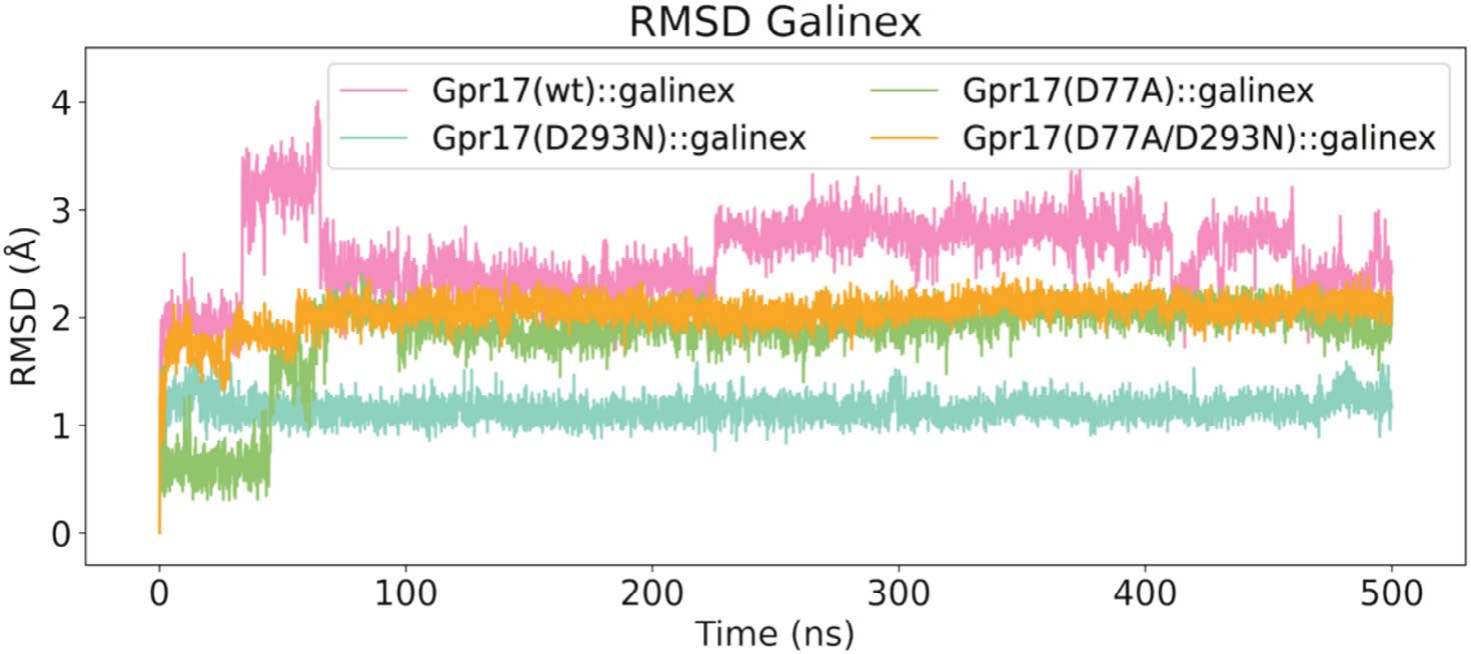
RMSD computed on the atoms of the ligand galinex in the different simulations to assess eventual differences in its stability of the interaction with the receptor.

Significant differences were observed in the stability of the agonist across different GPR17 genotypes. In the *wild‐type* receptor, galinex showed high conformational freedom, leading to significant fluctuations in the RMSD profile. In contrast, the mutant receptors exhibited a more stable interaction with the agonist.

To better understand this phenomenon, we performed hydrogen bond analysis between the receptor and the agonist over the simulation time. This analysis indicated that the increased stability of galinex in mutant interactions could be attributed to the formation of a smaller number of more persistent hydrogen bonds. Notably, in the D293N mutant, the NH group of galinex formed a hydrogen bond with C157 oxygen in ECL2, persisting for approximately 80% of the simulation time. This bond appeared to limit the degrees of freedom of the agonist, reducing the number of possible conformations (Figure S2).

We also assessed system stability using RMSF, calculated on the backbone atoms of the protein. The RMSF profiles revealed that the most fluctuating regions were associated with the intracellular and extracellular loops, as well as the terminal regions of the protein. In contrast, the transmembrane regions displayed higher stability. ICL3 (connecting TM5 and TM6) and ECL3 (connecting TM4 and TM5) were identified as the regions with the highest degree of flexibility, reaching maximum RMSF values of 6 Å. The transmembrane regions, on the other hand, exhibited higher stability with RMSF values stabilizing below 2.5 Å. The C‐terminus portion of the receptor (amphipathic helix 8) reached the maximum RMSF value, in some cases reaching 8 Å (Figure S3).

Another analysis performed to assess system stability involved the calculation of the changes in SASA throughout the simulations. In all the examined systems, the SASA values remained consistently below 11 000 Å^2^ and exhibited minimal variations, indicating that the proteins conformations remained stable and did not undergo significant alterations during the simulations (Figure S4).

### 3.4 Solvent Analysis (3D‐RISM)

To investigate the role of sodium ions on GPR17 activity, we used the 3D‐RISM method within the Solvent Analysis application of the MOE software to compute density maps of sodium ions within the receptor. This analysis, performed on the medoids of the most representative cluster of each MD simulation, provided a detailed spatial representation of the ion distribution inside the receptor, specifically within the presumed binding site.

Our findings confirmed the presence of a predicted sodium ion binding site within the helical core of the *wild‐type* GPR17 receptor. The analysis revealed the presence of well‐defined density maps for Na^+^ near D77 and D293, indicating the possibility for the ions to interact with the receptor by establishing ionic interactions with the two acidic residues (Figure 5).

**FIGURE 5:**
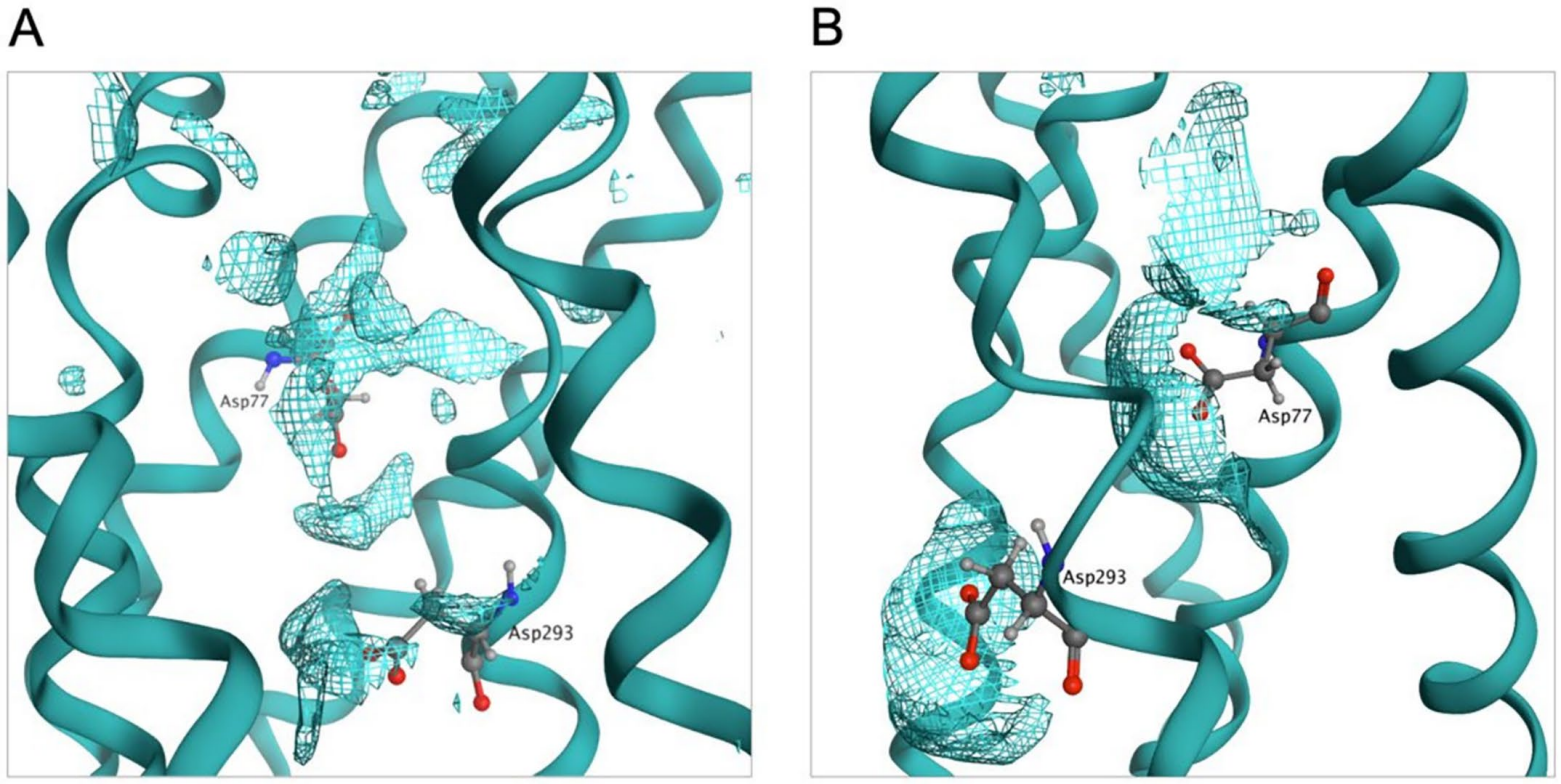
Sodium ions density maps around the residues D77 and D293 of GPR17^wt^ apo active (A) and apo inactive (B) conformations. The maps show higher dimensions in the inactive conformation of the receptor.

In the mutant receptors, on the other hand, the mutations in the residues that comprise the ion binding site led to a reduction in the dimensions of the density maps, thus impacting the ability of sodium to interact with these specific regions of GPR17.

The D77A mutation, which neutralizes the negative charge of aspartic acid in TM2 helix through alanine substitution, resulted in a moderate reduction in the overall density maps dimension, due to the absence of maps in proximity to D77. However, in the different D77A mutant conformations, the ion's ability to interact with the receptor is maintained by the presence of the *wild type* D293 residue (Figure 6).

**FIGURE 6:**
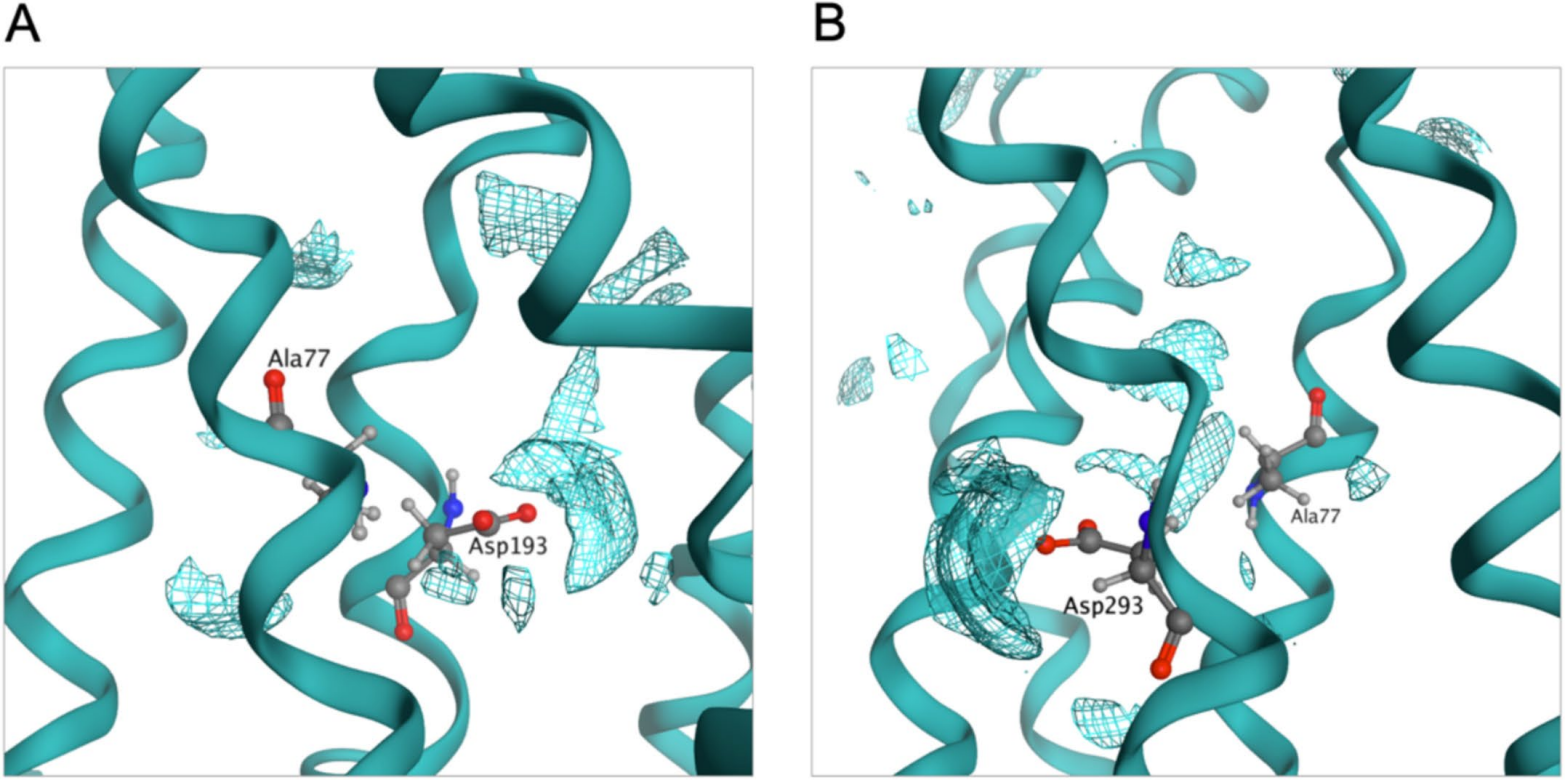
Sodium ions density maps around the residues A77 and D293 of the mutant GPR17(D77A) *apo* active (A) and *apo* inactive (B) conformations.

A more substantial reduction in the map dimensions was observed with the D293N mutant. This mutation not only reduced the ion probability of interacting with residue 293 in TM7 helix, but also affected its ability to interact with residue 77 in TM2 helix, thus indicating a major impact of this residue in the Na^+^‐GPR17 interaction (Figure 7).

**FIGURE 7:**
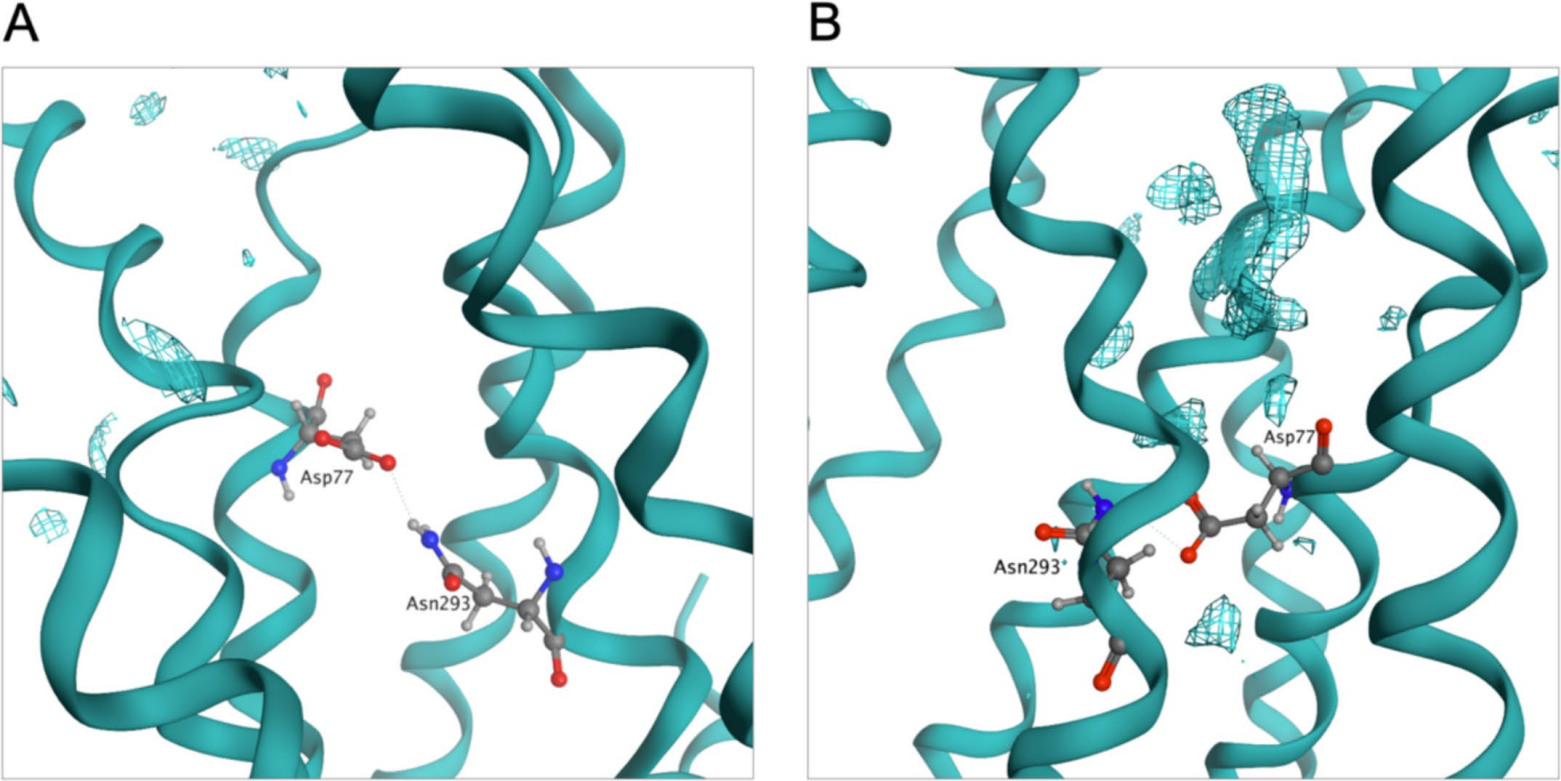
Sodium ions density maps around the residues D77 and N293 of the mutant GPR17(D293N) *apo* active (A) *apo* inactive (B) conformations.

Furthermore, comparing the results of the analyses performed on *wild type* GPR17 in active and inactive conformations, we noticed that the sodium density surfaces were reduced in the active receptor, indicating a lower likelihood of ion::receptor interaction.

This observation supports the hypothesis that the ion preferentially interacts with the inactive conformation of the receptor, whereas in the active conformation the structural rearrangements in the TM helices may lead to a reduction in the volume size of the binding pocket, thus preventing the interaction with the ion.

### 3.5 MD Simulations With Sodium Ions

Based on the results from Solvent Analysis, we manually placed a sodium ion in proximity to the detected density maps within different conformations of the *wild type* receptor. Subsequently, each structure, including the self‐activated *apo*, inactive *apo*, and active *holo* (bound to galinex) conformations, was subjected to a 250 ns MD simulation to investigate potential alterations in the receptor structure.

Our particular focus was on TM6 helix, which plays a critical role in Na^+^‐dependent effects on receptor activity. In this context, we computed the RMSD of the ⍺‐carbon atoms within TM6 helix. This analysis was performed both with and without sodium, allowing a comparison of the obtained data from both simulations (Figure S5).

Our simulations revealed that the presence of sodium ions maintains the stability of TM6 helix, as indicated by consistently low RMSD values (below 3 Å) throughout the simulation period. However, upon examining the RMSF, we observed that TM6 helix residues in the *apo* inactive state of GPR17 exhibited restricted mobility in the presence of sodium, in contrast to the higher values observed in simulations performed without it (Figure S6).

This finding supported the mechanism by which the ion stabilizes the inactive conformation of the receptor.

### 3.6 [ ^3^H]27‐Hydroxycholesterol Binding Assay

To investigate if the GPR17 variants mutagenized on sodium sites influence the binding properties of the receptor itself, we evaluated the ability of a GPR17 agonist (i.e., Asinex1) and of a receptor antagonist (i.e., Cangrelor) to displace [^3^H]27‐hydroxycholesterol binding to the selected receptor variants (i.e., D77A, D293N and D77A/D293N).

As depicted in Figure S7A, the radiolabeled hydroxycholesterol showed a rapid association kinetic, displaying a half‐time of 35.54 min. [^3^H]27‐Hydroxycholesterol was demonstrated to bind specifically 1321 N1 cells transfected with WT GPR17, because no significant specific binding was evidenced in 1321 N1 cells transfected with an empty vector (data not shown). Saturation experiments in WT GPR17 transfected cells, using different radioligand concentrations, revealed a Kd value of 7.05 ± 0.55 nM (Figure S7B), comparable to EC_50_ value obtained in GTPγS on the same cells.

Such binding protocol was used to characterize the pharmacological profile of GPR17 ligands toward the different GPR17 constructs profile. Empty vector transfected cells were run in parallel as negative controls and did not show significant specific binding (data not shown), confirming that the signal reported derives from GPR17‐expressing membranes and not from endogenous receptors.

In WT GPR17 transfected cells, Asinex 1 and Cangrelor showed a Ki of 297 ± 22 pM and 654 ± 74 nM, respectively, as previously reported. The obtained Ki values for Asinex 1, tested in the different GPR17 constructs, did not significantly differ from that obtained in the wild‐type form of the receptor (Figure 8 and Table 2). Similarly, in the [3H]27‐hydroxycholesterol binding assay, Cangrelor evidenced comparable Ki values for all the tested GPR17 receptor variants (Figure 8 and Table 2).

**FIGURE 8:**
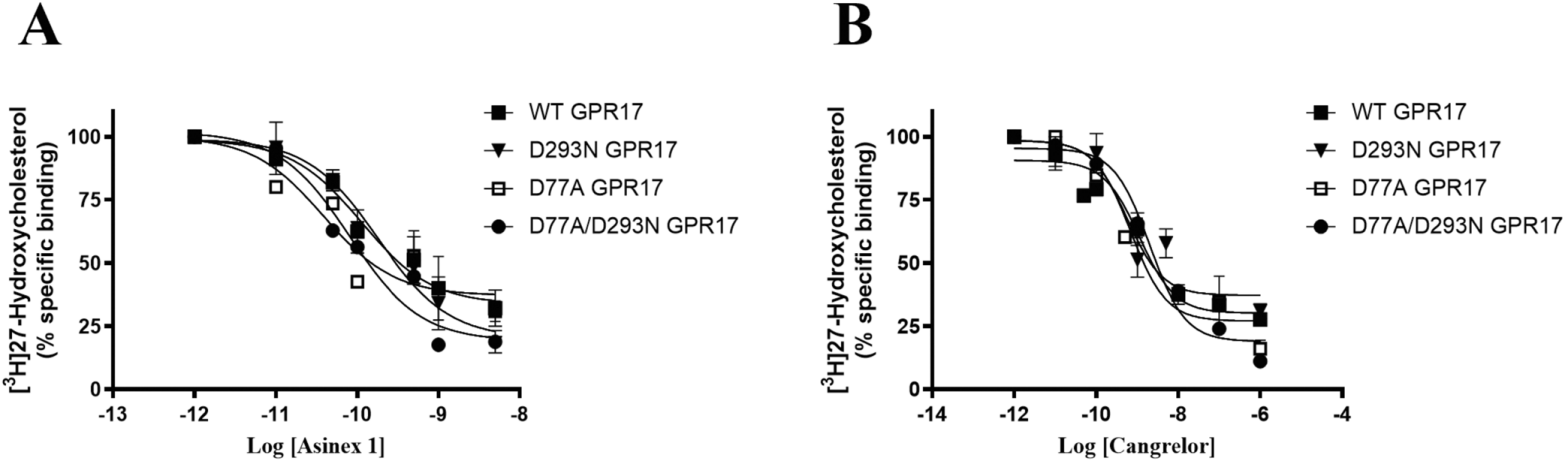
Displacement curves of Asinex1 (A) and Cangrelor (B) in the [^3^H]27‐Hydroxycholesterol binding assay. One thousand three hundred and twenty one N1 cells were transfected with WT GPR17, D77A, D293N, or D77A/D293N GPR17 constructs. Then, cells were incubated with the indicated concentrations of Asinex1 (A) or Cangrelor (B) in the presence of the radiolabeled [^3^H]27‐hydroxycholesterol. The 100% reference corresponds to the DPM signal associated with [^3^H]27‐hydroxycholesterol, measured in GPR17‐transfected cells not exposed to any unlabeled (cold) ligand. Values are expressed as mean ± SEM of three independent experiments, each performed in duplicate.

**TABLE 2:** Affinity binding values (Ki) of Asinex1 and Cangrelor obtained in [^3^H]27‐Hydroxycholesterol binding assay.

	WT GPR17	D77A GPR17	D293N GPR17	D77A/D293N GPR17
Asinex 1 (Ki, pM)	297 ± 22	389 ± 42	202 ± 23	216 ± 29
Cangrelor (Ki, pM)	654 ± 74	436 ± 48	746 ± 81	564 ± 64

*Note:* All data are mean ± SEM of three different experiments, each performed in duplicate.

Overall, these data suggest that the mutations in the sodium binding site did not significantly affect either the agonist or antagonist binding to the receptor.

### 3.7 [ ^35^S] GTPɣS Binding Assay on GPR17 Constructs

To evaluate if the GPR17 mutations in the putative sodium‐binding sites can affect receptor coupling to the G protein, as the result of the binding of the ligand to the receptor, the receptor variants were tested by the mean of the [^35^S]GTPɣS binding assay. The [^35^S] GTPɣS assay determines the level of G protein activation after the stimulation of the GPCR by an agonist, quantifying the binding of the radiolabeled non‐hydrolysable analogue of GTP to the Gα subunit [18].

Empty‐vector transfected membranes were run in parallel as negative controls and did not show significant specific binding or agonist‐induced stimulation (data not shown), confirming that the signal derives from GPR17‐expressing membranes and not from endogenous receptors.

Under sodium‐free conditions, any efficient agonist‐dependent G protein activation above unstimulated controls was detected in WT GPR17‐transfected membranes, indicating that removal of extracellular sodium prevents productive receptor‐G protein coupling in our system (Figure S8).

In our hands, the above‐mentioned GPR17 variants, mutated in the putative sodium‐binding sites, were all functional and coupled to G proteins.

In the presence of 150 mM of sodium, the GPR17 agonists that is, UDP‐glucose, LTD_4_ and Asinex1, showed potency values (EC_50_) of 9.62 ± 0.31 μM, 4.85 ± 0.32 nM and 550 ± 62 pM, respectively, when tested on WT GPR17 (Figure 7 and Table 2). These values are comparable to those reported previously in similar experimental conditions [1, 19].

Next, we compared the ability of GPR17 ligands to stimulate GTPγS binding in the different receptor variants. At 150 mM sodium concentration, the GPR17 receptor variants D77A, D293N, and D77A/D293N showed significantly lower EC_50_ values for Asinex1 with respect to the WT form (Figure 9, Table 3). Similar results were obtained for LTD_4_ (Figure 9, Table 3). In contrast, UDP‐glucose showed a significantly lower EC_50_ value with respect to the WT GPR17 in the D293N receptor variant only (Figure 9, Table 3), suggesting that sodium can differently modulate the purinergic and leukotriene binding site.

**FIGURE 9:**
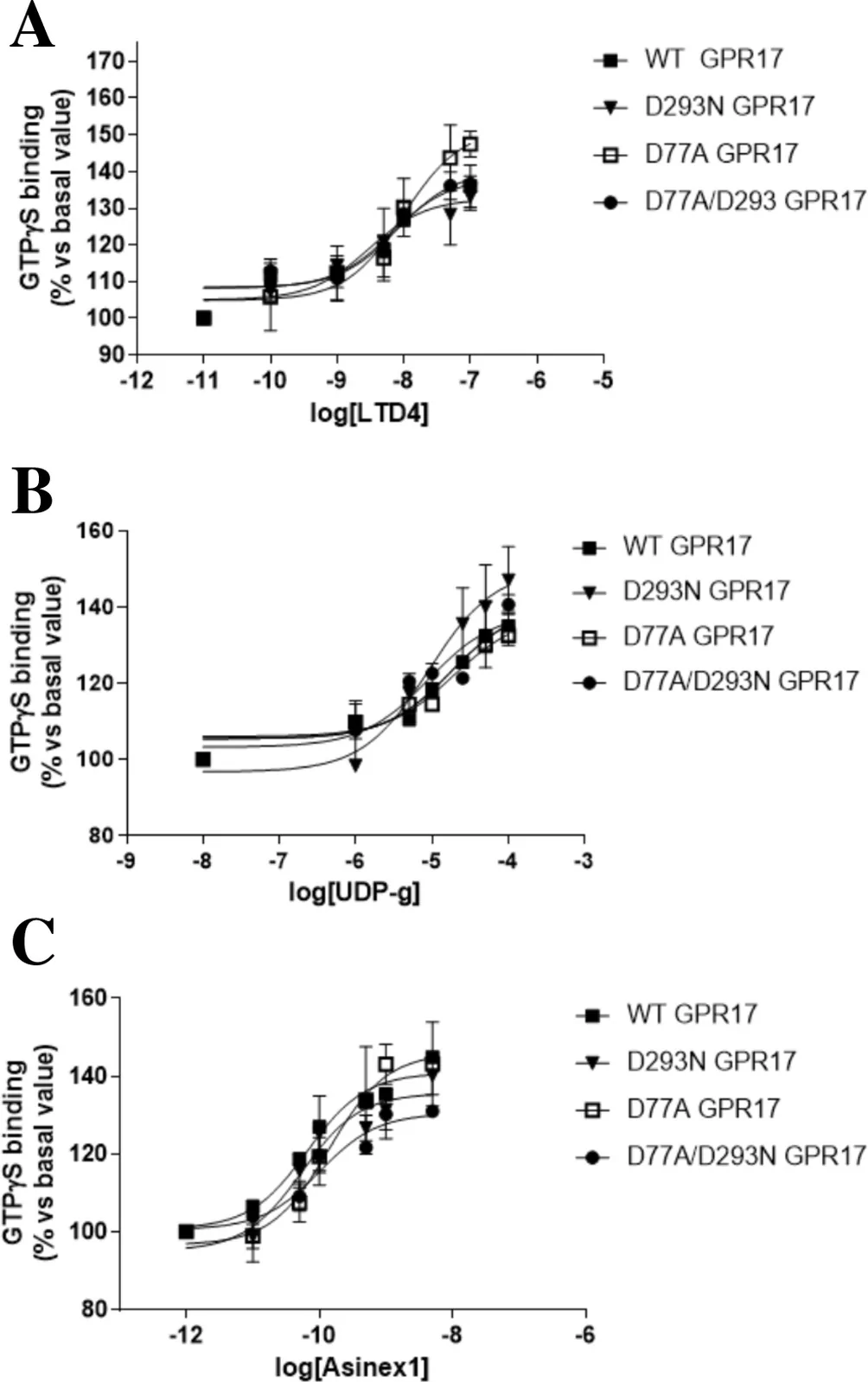
Dose–response curves of GPR17 agonists assessed by [^35^S]GTPγS binding assay. Membrane aliquots, obtained from 1321 N1 cells transfected with the WT GPR17, or with D77A, D293N or D77A//D293N GPR17 variants, were incubated with the indicated concentrations of LTD_4_ (A), UDP‐glucose (B), or Asinex1 (C), in the presence of 150 mM sodium, and the GTPγS binding assay was performed as described in the Methods section. All data were normalized to the basal radioactivity signal measured in membranes from GPR17‐transfected cells that were not exposed to any ligand; this unstimulated DPM value was set to 100% in each independent experiment. Values are expressed as mean ± SEM of three independent experiments, each performed in duplicate.

**TABLE 3:** EC_50_ values for Asinex 1, UDP‐glucose and LTD_4_ found in the GTPγS binding assay, at 150 mM sodium concentration, using membrane aliquots obtained from the different GPR17 variants (WT, D77A, D293N and D77A/D293N GPR17).

Agonist	WT GPR17	D77A GPR17	D293N GPR17	D77A/D293N GPR17
Asinex1	334 ± 35 pM	150 ± 20 pM*	101 ± 62 pM*	99 ± 62 pM**
LTD_4_	5.24 ± 0.48 nM	3.12 ± 0.62 nM*	2.94 ± 0.24 nM**	3.39 ± 0.32 nM*
UDP‐glucose	9.62 ± 0.31 μM	8.98 ± 0.91 μM	6.01 ± 0.51 μM*	8.95 ± 0.67 μM

* *p* < 0.05.

** *p* < 0.01 with respect to wild‐type GPR17.

### 3.8 cAMP Accumulation Experiments

In order to further investigate GPR17 functionality, GPR17 ligand (Asinex1, UDP‐glucose, and LTD_4_) ability to counteract Forskolin‐induced cAMP production was evaluated by radioactive cAMP assay.

In WT GPR17 transfected cells, Asinex1, UDP‐glucose and LTD_4_ showed EC_50_ values of 352 ± 30 pM, 5.23 ± 0.30 μM and 7.05 ± 0.60 nM, respectively, as previously reported [6, 20]. In GPR17 mutagenized variants, Asinex 1 displayed a significant enhancement in the potency value in D77A, D293N, and D77A/D293N GPR17 with respect to WT receptor form (Figure 10, Table 4). Similar results were obtained with UDP‐glucose or LTD_4_, thus suggesting that mutations in sodium binding site enhanced ligand potency in stimulating GPR17 functional response.

**FIGURE 10:**
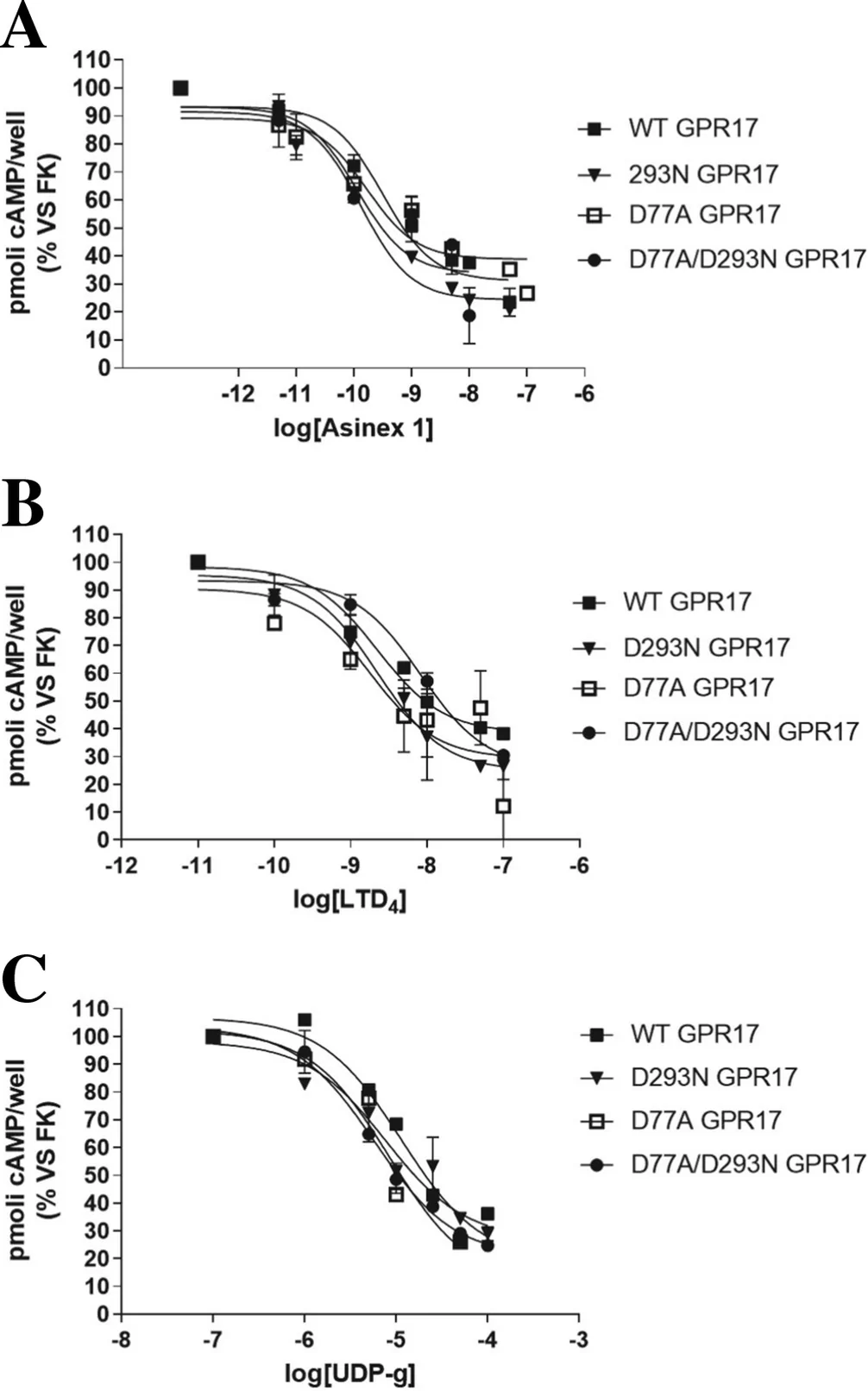
Dose–response curves of GPR17 ligands in the cAMP assay. One thousand three hundred and twenty one N1 cells transfected with the WT GPR17, or with D77A, D293N, or D77A/D293N GPR 17 variants were incubated with forskolin in the absence or presence of increasing concentrations of Asinex1 (A), LTD_4_ (B), or UDP‐glucose (C). The data are expressed as percentage of the total picomoles of cAMP produced by forskolin, set to 100%. The data are the result of three separate experiments; each performed in duplicate.

**TABLE 4:** EC_50_ values for Asinex 1, UDP‐glucose and LTD_4_ obtained in the cAMP assay.

	WT GPR17	D77A GPR17	D293N GPR17	D77A/D293N GPR17
Asinex 1	0.395 ± 0.40 nM	0.234 ± 0.029 nM*	0.223 ± 0.021 nM*	0.185 ± 0.020 nM*
LTD_4_	7.05 ± 0.60 nM	2.02 ± 0.30 nM*	4.32 ± 0.51 nM*	4.09 ± 0.52 nM*
UDP‐glucose	5.93 ± 0.30 μM	2.90 ± 0.21 μM*	3.15 ± 0.22 μM*	1.29 ± 0.22 μM*

* *p* < 0.05 with respect to wild‐type GPR17.

### 3.9 Design of an Optimized GPR17 Variant for Biochemical and Structural Studies

Based on the results described above, we designed an optimized variant of GPR17 for large scale expression and purification in insect cells. We selected a construct lacking amino acids 1–15 and with the thermostabilized moiety, b562 RIL (BRIL) in this case, inserted into the third intracellular loop (GPR17‐BRIL 16–339), like what has been done for other GPCRs [21]. In addition to a standard C‐terminal His tag, the construct was further modified with the insertion of an N‐terminal StrepTag for a more selective and efficient purification by affinity chromatography, and the potential glycosylation sites (N176 and N254) were mutated to Gln. Furthermore, we introduced the D293N mutation because, although it slightly reduces ligand binding and receptor coupling to G protein, it has been shown to produce a stabilizing effect on other P2Y receptors [12, 22]. The final construct is shown in Figure 8a. The engineered GPR17‐BRIL 16–339 construct was designed primarily to enhance receptor expression, conformational homogeneity, and stability for biochemical and structural applications, following established GPCR engineering strategies. In this construct, the thermostabilized BRIL moiety replaces the third intracellular loop (ICL3), which in native GPCRs plays a critical role in G protein recognition and productive coupling. Therefore, although suitable for structural and biophysical characterization, this engineered receptor is not expected to fully reproduce native G protein activation and downstream signaling. For this reason, functional sodium‐dependent effects on receptor–G protein coupling were assessed using full‐length signaling‐competent constructs expressed in mammalian cells.

The construct was successfully expressed with good yield in insect cells where it was effectively solubilized from the membrane fraction under mild detergent conditions (0.5% β‐DDM) (Figure 11b). We then performed mid‐scale (100–200 mL of cell culture) preparations to optimize the purification protocol. Solubilized membranes were applied to a Strep‐TactinTX column, and the receptor was then eluted with biotin (Figure 11c). Further purification by IMAC yielded highly pure preparations (Figure 11d), suggesting a promising starting point for large‐scale expression testing and production scale‐up.

**FIGURE 11:**
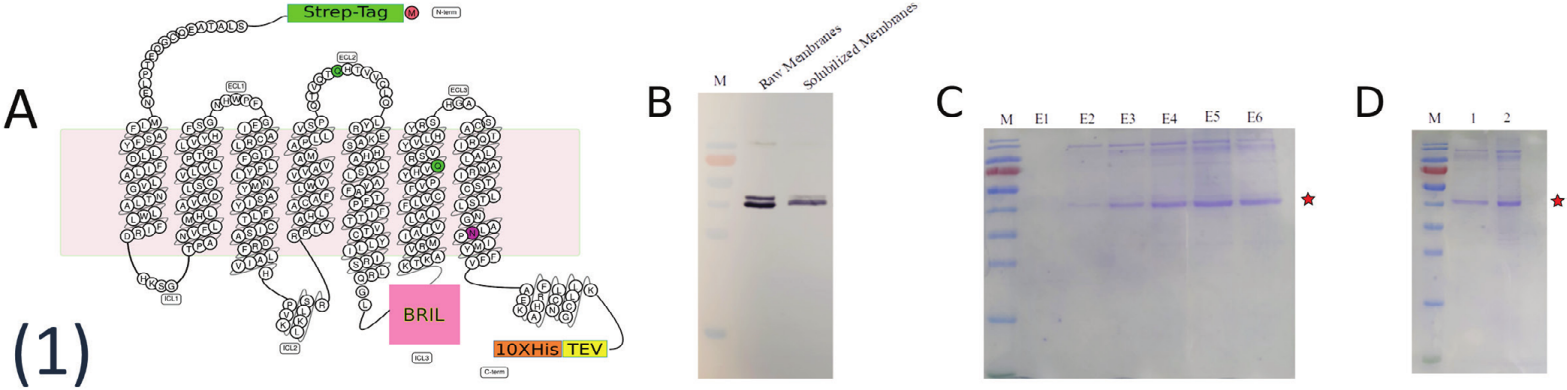
(A) Snake plot of the optimized GPR17 construct for expression in insect cells. The N‐ and C‐terminal tags are shown in green and orange, respectively. The BRIL moiety is colored in pink and the mutated residues are highlighted in green and purple. (B) Western blot analysis with anti‐His antibody of raw membrane and detergent‐solubilized fractions. (C) SDS‐PAGE of Strep‐TactinTX affinity purification. Fractions eluted with biotin are numbered from 1 to 6. (D) SDS‐PAGE showing the receptor after IMAC purification [1] and in the final concentrated preparation.

## 4 Discussion

GPR17 is a class‐A GPCR of increasing pharmacological interest due to its role in oligodendrocyte maturation and response to ischemic injury. While recent cryo‐EM studies have provided high‐resolution structural insights into its active conformation, the mechanisms regulating its conformational transitions remain poorly understood. Among class‐A GPCRs, sodium ions have emerged as key allosteric modulators, often stabilizing the inactive state via conserved binding sites. However, the extent and specificity of this effect vary depending on the receptor and the nature of the residues forming the sodium pocket.

In this work, we systematically investigated the role of sodium ions in modulating GPR17 conformational states and signaling output by combining molecular modeling, MD simulations, solvent mapping, and in vitro functional assays. Our computational results demonstrate that Na^+^ preferentially stabilizes the inactive conformation of GPR17, as evident from higher sodium density near the canonical binding site (D77/D293) and reduced TM6 flexibility in the presence of the ion. These observations are in line with established models in other GPCRs, where sodium occupancy correlates with stabilization of the inactive state by constraining the movement of key helices, particularly the TM6 one.

Interestingly, the mutagenesis of D77A (D^2.50^) and D293N (D^7.49^) had differential effects on sodium binding and receptor activity. While both residues contribute to sodium coordination, the D293N mutation caused a more significant disruption of the sodium binding site, as evidenced by the reduction of 3D‐RISM sodium density maps even at a certain distance from the considered residues. This suggests a more pivotal role of D^7.49^ in GPR17 compared to other class‐A GPCRs, where this position is typically occupied by asparagine. The unique presence of an acidic residue at this site in GPR17, a relatively rare feature among class‐A receptors (approx. 7%), may confer an enhanced sensitivity to sodium modulation.

From a broader point of view, our findings raise the possibility that sodium sensitivity may vary significantly even within class‐A GPCRs, depending on the specific combination of residues at conserved allosteric sites. While the presence of D^2.50^ is well established as essential for sodium binding in many receptors, our data suggest that D^7.49^, though less frequently conserved, can substantially modulate the sodium effect, particularly in receptors like GPR17 that possess both acidic residues. This combinatorial contribution should be considered in future studies of GPCR allostery and could be exploited for the design of ligands that selectively modulate receptor states by targeting these auxiliary sites.

In parallel to modeling studies, the role of sodium on GPR17 binding properties and functional responses was tested in vitro *using* receptor variants, mutagenized on sodium sites D77 and D293. All the GPR17 selective ligands showed comparable binding properties to GPR17 in mutated variants with respect to the wild‐type receptor. In contrast, a significant increase in receptor coupling to G proteins was noticed for the receptor ligand Asinex1 and LTD_4_ in the receptor variants D77, D293, and D77/D293 with respect to the wild‐type GPR17.

Notably, MD simulations and functional assays emphasize different physical aspects of the same allosteric architecture. In our simulations, D293 behaves as a key node of the extended polar/water network shaping cavity hydration and geometry; neutralizing D293 therefore produces larger local reorganizations within the helical core on the simulated timescale. Conversely, D77 represents the canonical electrostatic anchor of the sodium site in class‐A GPCRs, and its neutralization can strongly impair Na + occupancy and electrostatic “gating” of allosteric modulation. Functional readouts such as [35S]GTPγS binding and cAMP accumulation can amplify modest changes in coupling efficiency or basal equilibrium into comparatively large potency shifts, whereas standard MD observables capture local structural rearrangements that do not necessarily scale linearly with signaling output. Thus, D293N can appear “more disruptive” structurally, while D77A can appear “more impactful” functionally, reflecting complementary contributions of cavity architecture vs. electrostatic triggering. Moreover, the cryo‐EM template (PDB ID: 7Y89) lacks an unresolved segment at the N‐terminus (~50 residues), likely corresponding to a flexible/disordered region. Our conclusions mainly concern the transmembrane helical core and the conserved sodium pocket centered on D77/D293 and its coupling to intracellular activation microswitches; the missing N‐terminal segment is extracellular and spatially distant from this cavity. Therefore, it is unlikely to qualitatively alter sodium‐pocket geometry on the simulated timescales, although it may contribute to extracellular vestibule dynamics and ligand access in longer‐timescale or ligand‐entry focused studies. These data suggest that the loss of sodium‐mediated stabilization of the inactive state facilitates receptor activation and signal propagation, further supporting the role of sodium as a negative allosteric modulator of GPR17.

In the cAMP accumulation assay, all ligands showed a significant increase in potency in the sodium‐site mutants compared to WT, consistent with a reduced stabilization of the inactive state and a shift of the conformational equilibrium toward more readily activatable receptor ensembles. Sodium negative allostery has been reported for several class‐A GPCRs, including the ghrelin receptor (GHSR), where sodium occupancy shifts the conformational equilibrium toward inactive ensembles and reduces basal and agonist‐induced G protein activation. In that receptor, sodium modulation is attributed to the canonical class‐A sodium pocket rather than to uncommon substitutions at position 7.49. In contrast, GPR17 contains an acidic residue at 7.49 (D293), a relatively rare feature among class‐A GPCRs, which may strengthen or reshape the local hydrogen‐bond/water network and thereby tune sodium sensitivity [23].

In contrast, in different studies the removal of extracellular Na^+^ results in a decrease in the potency of ACh toward the M2R.

Moreover, the increased functional responsiveness observed in the mutants, particularly D293N, has implications beyond basic pharmacology. From a structural biology perspective, receptors that naturally adopt a more active conformation in the absence of sodium may be more suitable for stabilization, purification, and crystallization in agonist‐bound forms.

Accordingly, we engineered a BRIL‐tagged GPR17 construct incorporating the D293N mutation and achieved successful expression and purification in insect cells, providing a foundation for future structural and biophysical studies. It should be noted, however, that in this construct the third intracellular loop (ICL3) is replaced by BRIL to enhance stability and crystallizability. Because ICL3 is a key structural element for productive G protein engagement in class‐A GPCRs, this engineered receptor is not expected to fully reproduce native signaling properties. Consequently, sodium‐dependent functional effects on G protein coupling are more appropriately investigated in cell‐ or membrane‐based systems expressing the full‐length receptor, as performed in the present study.

Nevertheless, the purified construct represents a valuable tool for sodium‐dependent analyses at the level of ligand binding and conformational stabilization. In particular, it may enable in vitro assessment of sodium effects on ligand‐binding equilibria and receptor stability (e.g., thermostability or ligand‐induced stabilization assays), and could facilitate future structural determination of distinct conformational states under controlled ionic conditions.

## 5 Conclusions

This study supports a model in which sodium acts as a negative allosteric modulator of GPR17 by stabilizing inactive‐like receptor ensembles through the conserved sodium pocket centered on D77 (2.50) and the atypical acidic residue D293 (7.49). Computational solvent mapping and MD simulations indicate preferential sodium occupancy in inactive conformations and reduced TM6 mobility in the presence of the ion, consistent with canonical class‐A GPCR mechanisms. Mutations disrupting this pocket do not measurably alter ligand binding affinity, yet they increase agonist potency in G protein coupling and cAMP assays, consistent with a reduced stabilization of inactive conformations and an easier transition toward activatable states.

Direct experimental probing under sodium‐free conditions shows that removal of sodium abolishes detectable agonist‐driven G protein activation in WT membranes in our system, supporting the functional relevance of sodium for productive coupling under our assay conditions. Together, these results suggest that engineering sodium‐pocket variants can bias the conformational landscape of GPR17 toward signaling‐competent states, providing a rationale for construct design aimed at biochemical stabilization and structural studies. Future work should address slower conformational transitions on longer timescales and extend functional profiling to additional pathways (e.g., β‐arrestin recruitment/internalization) and to receptor–receptor crosstalk contexts.

## Author Contributions

**Luca Palazzolo:** writing – original draft, investigation, formal analysis, data curation. **Lara Russo:** methodology, formal analysis. **Davide Bianchi:** methodology, formal analysis. **Simona Daniele:** writing – original draft, investigation, formal analysis, data curation. **Ivano Eberini:** writing – review and editing, supervision, resources. **Maria Letizia Trincavelli:** writing – review and editing, supervision, funding acquisition. **Stefano Capaldi:** investigation, methodology, formal analysis.

## Funding

This work was supported by the Fondazione Italiana Sclerosi Multipla (2018/R/21), the MUR ‐ Ministero dell'Università e della Ricerca (Progetto Eccellenza 2023–2027), and the Università degli Studi di Milano (Azione A (PSR2023)).

## Conflicts of Interest

The authors declare no conflicts of interest.

## Supporting information


**Figure S1:** Ramachandran plot computed on GPR17 in active apo conformation (A) and inactive apo conformation (B). The only outlier values are associated to regions of the protein characterized by high flexibility: Ser90 is located in ICL1, while Tyr213 in ECL2.
**Figure S2:** Hydrogen bonds analysis between galinex and the different GPR17 genotypes over the simulation time.
**Figure S3:** Root mean square fluctuation (RMSF) computed on the backbone atoms of the receptor. The different regions of the protein are highlighted, showing how the intracellular (ICLs) and extracellular (ECLs) loops exhibit a higher flexbility with respect to the transmembrane regions.
**Figure S4:** Solvent accessible surface area (SASA) computed on the different receptor conformations and genotypes.
**Figure S5:** RMSD computed on the α‐carbons of transmembrane helix 6 (TM6) in the simulations performed, respectively, without sodium in the binding site (on the left) and with sodium (on the right).
**Figure S6:** RMSF computed on the backbone atoms of GPR17 during the different MD simulations. For each plot, the TM6 region is highlighted in pink (respectively, light pink for the simulations performed without sodium in the binding site, and dark pink in presence of the ion).
**Figure S7:** (A) Association curve of [^3^H]27‐Hydroxycholesterol binding (0.5 nM) to 1321 N1 WT GPR17 cells. (B) Saturation curve for specific [3H]27‐Hydroxycholesterol binding to 1321 N1 WT GPR17 cells. Saturation experiments were performed at 25°C (120 min). The data represent the mean ± SEM of at least two independent experiments, each performed in duplicates.
**Figure S8:** Dose–response curves of GPR17 agonists assessed by [35S]GTPγS binding assay. Membrane aliquots, obtained from 1321 N1 cells transfected with the WT GPR17 were incubated with the indicated concentrations of LTD4 or UDP‐glucose in the absence of sodium, and the GTPγS binding assay was performed as described in the Methods section. All data were normalized to the basal radioactivity signal measured in membranes from GPR17‑transfected cells that were not exposed to any ligand; this unstimulated DPM value was set to 100% in each independent experiment. The data are the mean ± SEM of three different experiments, each performed in duplicate.

## Data Availability

All relevant data will be deposited in the UniMi Dataverse at the following link: https://doi.org/10.13130/RD_UNIMI/SDVLAN.
