# Supplementary material for: Sodium Ions Affect GPR17 Conformational States and Functionality
Source: Proteins. 2026 Jul 12;94(12):1949–64. doi: 10.1002/prot.70157 (PMC13728507; doi:10.1002/prot.70157)
Supplement: Supplementary file 1 [file PROT-94-1949-s001.docx]

Supplementary materials

**Figure 1**: Ramachandran plot computed on GPR17 in active apo conformation (A) and inactive apo conformation (B). The only outlier values are associated to regions of the protein characterized by high flexibility: Ser90 is located in ICL1, while Tyr213 in ECL2.

**Figure 2**: Hydrogen bonds analysis between galinex and the different GPR17 genotypes over the simulation time.

**Figure 3**: Root mean square fluctuation (RMSF) computed on the backbone atoms of the receptor. The different regions of the protein are highlighted, showing how the intracellular (ICLs) and extracellular (ECLs) loops exhibit a higher flexbility with respect to the transmembrane regions.

**Figure 4**: Solvent accessible surface area (SASA) computed on the different receptor conformations and genotypes.

**Figure 5**: RMSD computed on the α-carbons of transmembrane helix 6 (TM6) in the simulations perfomed respectively without sodium in the binding site (on the left) and with sodium (on the right).

**Figure 6**: RMSF computed on the backbone atoms of GPR17 during the different MD simulations. For each plot, the TM6 region is highlighted in pink (respectively light pink for the simulations performed without sodium in the binding site, and dark pink in presence of the ion).

**Figure 7**: (A) Association curve of [^3^H]27-Hydroxycholesterol binding (0.5 nM) to 1321N1 WT GPR17 cells. (B) Saturation curve for specific [3H]27-Hydroxycholesterol binding to 1321N1 WT GPR17 cells. Saturation experiments were performed at 25 °C (120 min). The data represent the mean ± SEM of at least two independent experiments, each performed in duplicates.

**Figure 8:** Dose-response curves of GPR17 agonists assessed by [35S]GTPγS binding assay. Membrane aliquots, obtained from 1321N1 cells transfected with the WT GPR17 were incubated with the indicated concentrations of LTD4 or UDP-glucose in the absence of sodium, and the GTPγS binding assay was performed as described in the Methods section. All data were normalized to the basal radioactivity signal measured in membranes from GPR17‑transfected cells that were not exposed to any ligand; this unstimulated DPM value was set to 100% in each independent experiment. The data are the mean ± SEM of three different experiments, each performed in duplicate.
